# Could olfactory identification be a prognostic factor in detecting cognitive impairment risk in the elderly?

**DOI:** 10.1007/s11357-023-00779-5

**Published:** 2023-04-21

**Authors:** Alice Helena Delgado-Lima, Jaime Bouhaben, Sergio Martínez-Zujeros, Beatriz Pallardo-Rodil, Javier Gómez-Pavón, María Luisa Delgado-Losada

**Affiliations:** 1 Experimental Psychology, Cognitive Processes and Speech Therapy Department, Faculty of Psychology, Complutense University of Madrid, 28223 Pozuelo de Alarcón, Spain; 2 Geriatric Department, Hospital Central de La Cruz Roja “San José Y Santa Adela”, 28003 Madrid, Spain; 3 Group of Neurosciences: Psychoneuroendocrinology, Neuroimaging and Molecular Genetics in Neuropsychiatric Diseases, Instituto de Investigación Sanitaria San Carlos (IdISSC), Hospital Clínico de Madrid, 28040 Madrid, Spain

**Keywords:** Olfactory dysfunction, Odor identification deficits, Cognitive decline, Aging

## Abstract

Aging contributes to the deterioration of the olfactory system in humans. Several studies indicate that the olfactory identification test alone may function as a screening test for olfactory dysfunction and they are more feasible to apply in clinical practice. Olfactory identification may be a predictor for cognitive impairment. Multiple studies have considered the use of odor identification as a measure to identify the conversion from normality to mild cognitive impairment or dementia. The objectives were (i) to elucidate the associations between cognitive status and olfactory identification performance in aging; (ii) understand the predictive value of olfactory capacity in identifying subjects with cognitive impairment risk; and (iii) to study how cognitive status and olfactory identification relate with other variables of wellness in aging, such as functional capabilities and clinical measures. For this purpose, a group of 149 participants (77.15 ± 7.29 years; 73 women of 76.7 ± 8 years and 76 men of 77.6 ± 6.52 years) were recruited and were subjected to a sociodemographic questionnaire, a psychological screening tool of general cognitive status, an olfactory identification evaluation, and clinical measures. The participants were divided into groups based on their cutoff scores of previous scientific reports about the Spanish version of Montreal Cognitive Assessment. Our results indicate an age-associated decline in olfactory identification ability and intensity of odor perception. The predictive ability of olfactory identification scores for the risk of mild and severe impairment is around 80%. Olfactory identification decreases with cognitive function. Performance in odor identification is associated with impairment of episodic memory and executive functions. These findings further our current understanding of the association between cognition and olfaction, and support olfactory assessment in screening those at higher risk of dementia.

**Supplementary Information:**

The online version contains supplementary material available at 10.1007/s11357-023-00779-5.

## Introduction

Until today, the sense of smell is one of the least explored senses of human nature and much of its functions have yet to be clarified [1]. Nonetheless, other senses such as hearing or sight are routinely screened in clinical practice in order to detect issues that may impact quality of life, extension that is not bestowed to the olfactory sense, leading to a number of olfactory dysfunction to go unnoticed [2–4].

In patients with olfactory dysfunction (OD), an impact is usually observed on activities of daily living (ADLs), personal hygiene, safety, and sexual behavior [5, 6]. It has been suggested that the malnutrition associated with age is due OD alone or associated with taste alteration derived by retronasal olfactory alterations [7].

Less than a quarter of individuals with OD are conscious of their deficit until tested [8]. Olfactory capacity is evaluated through tests that measure threshold, discrimination, and identification of odors. Olfactory threshold represents the level of odor detection at low concentration, meaning the least detectable concentrations of odorant that can be perceived, whereas discrimination is the distinction of different odors, while identification refers to the ability to name or associate an odor [3, 9, 10].

Several studies indicate that the Olfactory Identification Test alone may function as a screening test for olfactory dysfunction or follow-up of olfactory function [11, 12], and they are more feasible to apply in clinical practice [13, 14]. Compared with other measures of olfactory performance, odor identification is a high-level cognitive operation, with greater cognitive load [15, 16]. A lot of evidence of test validity and reliability has been obtained in other cultures and languages. Including the Spanish population by Delgado-Losada et al. [17].

It is well established that normal aging is often accompanied by a decline in smell functioning [12, 18]. Epidemiological studies show that the prevalence and severity of olfactory dysfunction increase with age [4, 19–21]. Thus, 10% of people older than 65 years have some form of olfactory dysfunction ranging from mild loss to anosmia [8, 18, 22] affecting 62 to 80% of persons older than 80 years [20, 21, 23]. Olfactory loss is accompanied by structural abnormalities of the olfactory epithelium; the olfactory bulb and the central olfactory cortices [24, 25] found that the surface of the olfactory epithelium decreased during aging because of frequent presence of metaplastic respiratory epithelium, which could explain the age-related decline in olfaction. However, community studies have shown that olfactory impairment is associated with an increased incidence in cognitive impairment in the general population [26] suggesting that impaired olfactory functioning in older adults may not be due solely to changes in the respiratory epithelium.

Since 1999, Graves and colleagues, in a community-based study, showed that olfactory identification could be a predictor of cognitive decline [27], fact that have been elucidated by other investigations such as those by Schubet et al., Devand, and Kreisi [22, 26, 28]. Evidence of olfactory identification deficit in clinically normal elderly adults who go on to develop cognitive impairment, dementia, or AD has emerged in a number of large-scale studies. A large number of studies, which have been published in the last 5 years, demonstrate that odor identification discriminates between cognitively normal individuals, those with mild cognitive impairment (MCI), and those at risk for Alzheimer’s disease (AD). Some of these studies have considered the use of odor identification tests for the study of conversion from normality to MCI or dementia [22, 29, 30].

Longitudinal studies have shown that among cognitively normal individuals, those with poor odor identification scores at baseline are more likely to develop MCI than those with good odor identification scores [31–33] and individuals with MCI who have odor identification impairment are more likely to show progressive cognitive decline and convert to AD [34, 35].

Several studies have found that patients with the amnestic subtype of MCI show greater impairment in odor identification compared to non-amnestic MCI. Patients with multiple domain amnestic MCI have also been reported to show poorer olfactory function than patients with other subtypes, which suggests that those at highest risk of conversion from MCI to AD show the greatest impairment on olfactory testing [33, 36, 37]. In other studies, authors compared that odor identification test was used for clinical trials and reported that the sensitivity and specificity of such a test to detect conversion from amnestic MCI to AD were similar to those of more expensive and invasive markers, that is, somewhat inferior to structural MRI but similar to CSF biomarkers [34, 38].

Cross-sectional and longitudinal population-based studies have elucidated that olfactory identification deficits are associated with impairment in several cognitive domains mainly memory and executive functions [15, 39, 40].

The aims of this investigation was (i) to elucidate the associations between cognitive status and olfactory identification performance in aging; (ii) understand the predictive value of olfactory capacity in identifying subjects with cognitive impairment risk; and (iii) to study how cognitive status and olfactory identification relates with other variables of wellness in aging, such as functional capabilities and clinical measures.

## Method

### Participants

A total sample of 149 elderly participants (77.15 ± 7.29 years; 73 women of 76.7 ± 8 years and 76 men of 77.6 ± 6.52 years) were recruited from Geriatric Department from Hospital Central de la Cruz Roja “San José y Santa Adela” (Madrid, Spain) and Complutense University of Madrid. All participants were informed about the study guidelines and objectives and signed an informed consent prior to measures’ collections.

Inclusion criteria were (i) to be aged between 60 and 90 years; (ii) no prior diagnosis of dementia; (iii) no history of any neurological alterations, such as stroke, head trauma, and encephalitis; (iv) absence of current otorhinolaryngology alterations; and (v) compliance with testing procedures. Exclusion criteria were (i) medical history of olfactory alterations, including nasal polyposis, sinusitis, or prior nasal surgery; (ii) medication intake with repercussion in olfactory performance (such as some antibiotics, antiepileptics, antithyroids, benzodiazepines, or antiarrhythmics); (iii) presence or suspicion of psychiatric alterations, such as depressive or psychotic disorders (self-reported by the participant or present in clinical history); and (iv) presence of olfactory deficits or alterations due COVID-19 infection (self-reported or present in the clinical history).

From the total sample, a subsample of 122 participants (80.01 ± 8.83 years; 57 women of 80.6 ± 8.83 years and 65 men of 79.5 ± 8.87 years) also underwent clinical assessment (see section “Measures and procedure”). As this study section involved invasive tests (serological blood withdrawal), participation in clinical assessment was voluntary. Participation flow diagram is available at Supplementary Material S1.

### Measures and procedure

The assessment protocol was composed of a sociodemographic questionnaire, a psychological screening of general cognitive status, an olfactory evaluation, and clinical measures.

Sociodemographic *questionnaire*: A questionnaire survey was fulfilled by participants in order to collect sociodemographic and clinical information related to health, smoking habits, and prior olfactory status. Due to the health situation when data was collected, information about COVID-19 previous diagnoses was also obtained and analyzed.

*Global cognitive status*: Global cognitive function was assessed by Montreal *Cognitive Assessment (MoCA)* instrument [41]. This cognitive test covers many cognitive skills, scores range from 0 to 30, and cognitive impairment is defined by values < 26. This test assesses the main cognitive areas: immediate and delayed memory (free and cued recall), language, visuoperceptual and visuospatial capacities, motor planning, executive function, attention, and cognitive judgment. MoCA test is more specific to evaluate cognitive domains (attention, concentration, memory, language, calculation, orientation, and executive functions) and is considered the best test to detect mild cognitive impairment [42]. As a screening test, MoCA also provides cutoff points which may accurately guide in cognitive decline diagnosis. MoCA’s seminal work indicates 26 as the cutoff point between cognitive impairment and healthy aging [42], whereas other studies [43, 44] also establish 17 in order to discriminate more serious cognitive impairment. These cutoff points were adopted in the present study in order to interpret MoCA scores and describe categories, with no diagnostic meaning. Further, in subsample analyses, MoCA subscores for five cognitive domains were obtained: verbal fluency, short-term memory, conceptual thinking/abstraction, calculus, and spatial orientation. With a similar procedure, other investigations have used MoCA in other pathologies [45–48].

*Olfactory performance*: Olfaction was assessed with the Identification Smell Test, from Sniffin’ Sticks Olfactory Test. The original instrument (Burghart Messtechnik GmbH, Wedel, Germany) was adapted to the Spanish population by Delgado-Losada et al. [49]. Identification Smell Test was also adapted to the Spanish population as an independent instrument in Delgado-Losada et al. [17]. Cultural aspects can affect exposure and frequency of food and odors, and consequently familiarity with odors, leading to potential cultural bias in odor assessment. In order to obtain an accurate assessment of odor function, participants must be familiar with all descriptors used, which means that adaptation of odorants and distractors to the cultural environment is required [50, 51], such adaptations were made in Delgado-Losada [17]. This adaptation is a validated and extended procedure which allows to obtain three different olfactory identification (OI) measures: recognition score, free-recall score, and subjective intensity score (measured with a complementary visual analog scale which assesses the perceived intensity of each odorant). Administration procedure was validated in Delgado-Losada et al. [49]. Among the psychometric properties of Spanish adaptation [17, 45], reliability coefficients range between 0.56 and 0.91 (Cronbach’s alpha), internal validity is tested through confirmatory factor analysis, and test–retest correlation coefficient [0.69] shows proper stability across measures. Cultural adaptation of odor descriptors is also highlighted in these validation studies. In the present study, recognition and subjective intensity scores were obtained.

- *Recognition score*: This score indicates whether each odorant is correctly identified through a four-alternative forced-choice method. The odor pen is presented to the participant, and he or she has to recognize the target odor between four odor descriptors. Correct answers from the 16 items are added in order to calculate this score.
- *Subjective intensity score*: This score provides a subjective measure of odor intensity. After each pen presentation, subjective intensity of odorant is scored within a 1–10 visual analog scale. Subjective intensity score gives additional value to olfactory identification performance. The subjective intensity score is computed as the arithmetic mean of the intensity given to each item.

Olfactory assessment procedure was as follows. Identification Smell Test, from Sniffin’ Sticks Olfactory Test, is composed of 16 pens with a length of 14 cm and a diameter of 1.3 cm, being each pen filled with 4 ml of the corresponding liquid odorant. The evaluator takes the pen’s cap off and presents the tip of the pen to the participant’s nostrils for 3 s, with an approximate distance of 2 cm. In no case, the tip of the pen physically touches the participant’s nose.

*Serological and clinical assessment*: Blood work was obtained from a subsample of volunteer participants, and the following parameters were analyzed: albumin, vitamin D, cholesterol, lymphocytes, and vitamin B12 (fasting blood draw). Moreover, Barthel index and Function Ambulation Categories (FACs) were also obtained in this part of the evaluation. To evaluate functional capabilities, Barthel Index was used. Barthel Index consists of 10 items that measure a person’s daily function, specifically the ADLs and mobility. The items include feeding, moving from a wheelchair to bed and returning, grooming, transferring to and from the toilet, bathing, walking on a level surface, going up and down stairs, dressing, and continence of bowels and bladder. Barthel Index is scored from 0 to 100, with 0 point indicating complete care dependency [52]. While FAC is a functional walking test that evaluates ambulation ability. This 6-point scale assesses ambulation status by determining how much human support the patient requires when walking, regardless of whether or not they use a personal assistive device [53].

### Study design

The design for this investigation is a cross-sectional non-experimental design, as no manipulation for independent variables nor random allocation were performed. Assessment procedure took place between July and September of 2021. All participants were administered, in the first visit, the sociodemographic questionnaire, the Montreal Cognitive Assessment, and the Olfactory Identification Test (from Sniffin’ Sticks Olfactory Test). A subsample also underwent serological analyses, and indexes for health status were obtained (Barthel Index and FAC), which took place in the second visit. Blood withdrawal was voluntary, as some participants declined to participate in it (*n* = 27). Hence, this subsample (*n* = 122) underwent serological analyses and indexes for health status (second visit).

This study was ruled by the principles of the Declaration of Helsinki (Edinburgh, 2013) and was approved by the Ethics Committee from University Hospital San Carlos (Madrid, Spain) (ref. number 20/515-E). The study was adjusted to standards of good clinical practice (art.34 RD 223/2004; community directive 2001/20/CE), and to the protection of personal data and confidentiality (European Data Protection Regulation, and in accordance with the Organic Law 3/2018 on the Protection of Personal Data and Guarantee of Digital Rights).

### Statistical analyses

Firstly, descriptive analysis based on age was performed. Mean and standard deviation were calculated for each group of cognitive status. Due to the nature of the sample, differences in Age were expected; hence, Age is considered a covariable in every statistical analysis within this study.

Next, a two-way between-subject ANOVA model was adjusted for cognitive performance (MoCA) as a dependent variable, with olfactory status (*severe impaired olfaction, mild impaired olfaction and unimpaired olfaction*), age, and interaction Age × Olfactory status as independent variables. Percentiles 10 and 5 were used to assign patients to *mild impaired* and *severe impaired* categories, respectively. Therefore, the olfactory status variable was obtained from the Olfactory Identification Test result. These olfactory status’ categories are descriptive, and they have no diagnostic purpose. Age categories were established as [< 70, 70), [70, 80) and [80, > 80). Post hoc between-group multiple comparisons were performed under Tukey’s HSD test.

After that, participants were split into three performing categories according to their MoCA general score: severe cognitive impairment (SCI, MoCA score < 17), mild cognitive impairment (MCI, MoCA score between 17 and 26), and healthy controls (HC, MoCA score > 26). Then, logistic regression models were estimated in order to classify participants in their respective cognitive categories (HC vs MCI and HC vs SCI). This analysis was performed in order to study the classification power of olfactory identification score, and so its potential use in cognitive impairment diagnosis.

Finally, secondary analyses within the subsample who underwent serological and clinical assessment (*n* = 122, 80.01 ± 8.83 years; 57 women of 80.6 ± 8.83 years and 65 men of 79.5 ± 8.87 years) were performed. First, descriptive analysis with mean and standard deviation statistics was obtained. Later, linear regression models were estimated for each MoCA subscore, with age and olfactory performance variables as predictors (backward method). Finally, relationships between olfactory performance, cognitive performance, and other clinical variables were studied through Pearson’s correlations.

## Results

Descriptive analysis of the overall sample by cognitive status is shown in Table 1, whereas Table 2 shows descriptive analysis by age groups. As it was expected, the *Age* effect might be observed between cognitive status groups, so this has been taken into account in forward analyses. No differences regarding Sex, Allergies, COVID-19 prior diagnosis, Smoking, and Alcohol consumption were elucidated. There was also no evidence of differences between those participants who suffered from COVID-19 in the past and those who did not in olfactory performance nor cognitive performance. *t* tests were performed on Olfactory Identification-Recognition (*t* = 1.02, df = 61.86, *p* = 0.308), Olfactory Identification-Subjective intensity (*t* = 0.09, df = 55.61, *p* = 0.926) and MoCA score (*t* = 0.72, df = 54.91, *p* = 0.474).

**Table 1:** Descriptive analysis in overall sample by cognitive impairment risk

	Severe cognitive impairment	Mild cognitive impairment	Healthy controls		
*Sample size*	*44*	*55*	*46*		
	Mean (SD) or count	Mean (SD) or count	Mean (SD) or count	*F* or chi	*p*
Sex (women)	26	20	25	5.83	0.054
Age	81.4 (7.24)	77.5 (6.47)	72.6 (5.56)	20.85	< 0.0001**
Previous COVID-19 diagnosis	10	14	5	3.62	0.163
Allergies	1	7	8	5.65	0.059
Frequent smoking	2	3	0	2.48	0.288
Frequent alcohol consumption	0	0	0	-	-
Identification-Recognition	7.98 (2.37)	9.67 (2.44)	12.7 (2.05)	-	-
Identification-Subjective intensity	5.59 (1.1)	5.89 (1.79)	7.98 (2.51)	-	-
MoCA	12.6 (4)	21.6 (2.11)	27.3 (1.05)	-	-

^*^ *p* < 0.05, ** *p* < 0.01

**Table 2:** Descriptive analysis in overall sample by age groups

	< 70 years	70–80 years	> 80 years		
*Sample size*	*27*	*62*	*56*		
	Mean (SD) or count	Mean (SD) or count	Mean (SD) or count	*F* or chi	*p*
Sex (women)	17	32	27	1.61	0.446
Age	64.7 (3.24)	75.1 (2.83)	87.1 (3.86)	-	-
Previous COVID-19 diagnosis	10	23	24	5.83	0.055
Allergies	5	8	2	5.15	0.076
Frequent smoking	0	2	3	1.81	0.404
Frequent alcohol consumption	0	0	0	-	-
Identification-Recognition	11.1 (2.71)	10 (2.65)	8.46 (2.85)	9.63	0.0001**
Identification-Subjective intensity	6.77 (1.86)	6.61 (2.43)	5.63 (1.61)	4.42	0.014*
MoCA	24.5 (3.46)	21.4 (5.6)	15.2 (5.86)	-	-

^*^p < 0.05, ** *p* < 0.01

**Fig. 1:**
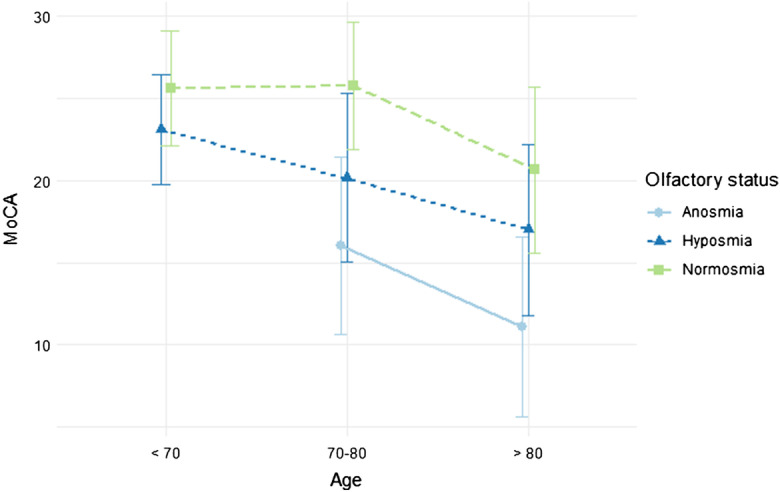
Mean graph of MoCA score by Age and olfactory status factors

ANOVA model on MoCA general score shows main effects of *Age* (*F* = 36.52, df = 2, *p* < 0.0001) and *Olfactory status* (*F* = 33.65, df = 2, *p* < 0.0001). There is no evidence to support *Age* × *Olfactory status* interaction effect (*F* = 0.825, df = 3, *p* = 0.48). These results may be found in Fig. 1. Post hoc comparisons on *Age* show significant differences between [< 70, 70) and [80, > 80) (dif = 8.162, *p* < 0.0001) and between [70, 80) and [80, > 80) (dif = 6.437, *p* < 0.0001). On the other side, post hoc comparisons on *Olfactory status* show significant differences between normosmic smell and hyposmia (dif = 4.083, *p* < 0.0001), between normosmic smell and anosmia (dif = 8.652, *p* < 0.0001), and between hyposmia and anosmia (dif = 4.569, *p* = 0.0005).

The ability of olfactory performance to distinguish between healthy participants and mild impaired participants (MoCA ≥ 26 vs MoCA between 18 and 25) and between mild impaired participants and severe impaired participants (MoCA between 25 and 18 vs MoCA ≤ 17) was tested with logistic regression models. In each table, model 1 refers to the baseline model, as it shows prediction performance with just *Age* as predictor. On the other side, model 2 involves the introduction of the two olfactory performance scores to test how much the model improves. Comparison between model 1 (baseline) and model 2 is performed with ANOVA with likelihood ratio test. Alpha is set at *α* = 0.05/2 (Bonferroni correction).

Table 3 shows results for healthy vs mild impaired participants (MoCA above or below 26, respectively). The inclusion of olfactory performance scores to the model significantly improves it (model 1 vs model 2, chi = 54.304, df = 2, *p* < 0.0001). Both olfactory scores are significant predictors within model 2.

**Table 3:** Logistic regression models (baseline or Model 1 and definitive or Model 2) of HC vs MCI

	Estimate	Error	*p*	Sensitivity	Specificity	AUC
*Model 1*						
Intercept	− 9.472	2.252	< 0.0001**	0.64	0.72	0.614
Age	0.133	0.03	< 0.0001**			
*Model 2*						
Intercept	1.288	3.118	0.679	0.79	0.87	0.821
Age	0.129	0.04	0.0012**			
Olfactory Identification-Recognition	− 0.694	0.133	< 0.0001**			
Olfactory Identification-Subjective intensity	− 0.417	0.135	0.0019**			

^*^p < 0.05, **p < 0.01

**Fig. 2:**
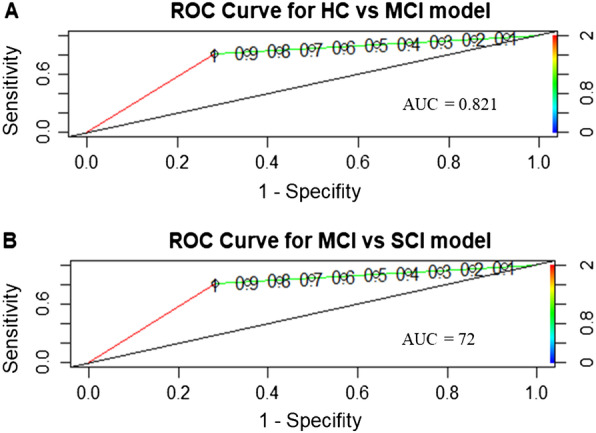
ROC curves for **A** HC vs MCI model and **B** MCI vs SCI model

Table 4, on the other hand, shows results for mild cognitive impaired vs severe cognitive impaired participants (MoCA above or below 18, respectively). Again, the inclusion of olfactory performance scores to the model significantly improves it (model 1 vs model 2, chi = 54.304, df = 2, *p* < 0.0001). However, subjective intensity score is not a significant predictor of cognitive status category (*p* = 0.083). ROC curves for both models may be checked in Fig. 2.

**Table 4:** Logistic regression models (baseline or model 1 and definitive or model 2) of MCI vs HCI

	Estimate	Error	*p*	Sensitivity	Specificity	AUC
*Model 1*						
Intercept	− 11.023	2.398	< 0.0001**	0.78	0.65	0.65
Age	0.129	0.03	< 0.0001**			
*Model 2*						
Intercept	− 3.323	2.8	0.235	0.83	0.69	0.72
Age	0.098	0.031	0.0021**			
Olfactory Identification-Recognition	− 0.388	0.09	< 0.0001**			
Olfactory Identification-Subjective intensity	− 0.261	0.151	0.083			

^*^p < 0.05, **p < 0.01

Finally, descriptive analysis of the remaining clinical variables is shown in Table 5.

**Table 5:** Descriptive analysis in subsample (*n* = 122)

	Mean (SD) or count
Sample size	122
Sex (women)	57 (46.72%)
Age	80.01 (8.83)
Identification-Recognition	8.84 (2.69)
Identification-Subjective intensity	5.67 (1.4)
MoCA—Total score	16.94 (5.91)
MoCA—Language score	1.88 (0.37)
MoCA—Short-term memory score	0.69 (1.21)
MoCA—Calculus score	1.19 (1.08)
MoCA—Abstraction score	1.47 (0.72)
MoCA—Orientation score	5.21 (2.32)
Barthel Index	71.48 (19.56)
FAC Index	3.06 (1.5)
Vitamin B12	541.6 (228.83)
Vitamin D	22.19 (12.43)
Albumin	3.3 (0.55)
Cholesterol	152.75 (41.02)
Lymphocytes	2.54 (6.85)

MoCA domain scores were obtained: Language, Short-term memory, Calculus, Conceptual thinking, and Spatial orientation. Linear regression models were performed for each subscore. Short-term memory is significantly predicted by age (*b* =  − 0.027, *t* =  − 2.297, *p* = 0.023) and olfactory performance (Olfactory Identification-Recognition, *b* = 0.139, *t* = 3.577, *p* = 0.0005) (see Fig. 2), whereas conceptual thinking is significantly predicted just by olfactory performance (Olfactory Identification-Recognition, *b* = 0.054, *t* = 2.18, *p* = 0.031). As variability in language score was so poor (max = 2, mean = 1.88, SD = 0.37), this subscore was dichotomized in two categories: 2 and below. Two samples independent *t* test was calculated (*t* = 1.412, df = 13.403, *p* = 0.181). Finally, simplified correlation matrix of Age, MoCA total score, and both olfactory performance scores with the remaining clinical variables might be found in Table 6. Complete correlation matrix is attached to Supplementary Material S2. Olfactory performance, represented by Olfactory Identification-Recognition score, correlates significantly with MoCA (*r* = 0.472, *p* < 0.0001) and the Barthel Index (*r* = 0.261, *p* = 0.0078), whereas MoCA score correlates with age (*r* =  − 0.258, *p* = 0.0049) and also the Barthel Index (*r* = 0.384, *p* < 0.0001). Non-significant but relevant correlation was also found between Olfactory Identification-Recognition and FAC Index (*r* = 0.195, *p* = 0.061).

**Table 6:** Simplified Pearson’s correlation matrix with clinical variables on subsample (*n* = 122)

	Age	Identification-Recognition	Identification-Subjective intensity	MoCA
Age	-			
Identification-Recognition	− 0.032	-		
Identification-Subjective intensity	0.02	0.123	-	
MoCA	− 0.258**	0.472**	0.147	-
Barthel Index	− 0.295**	0.261*	0.116	0.384**
FAC Index	− 0.239	0.195	0.102	0.192
Vitamin B12	0.052	− 0.114	− 0.037	− 0.012
Vitamin D	0.131	− 0.054	0.105	− 0.061
Albumin	− 0.113	0.081	− 0.026	0.035
Cholesterol	− 0.326**	− 0.024	0.067	0.083
Lymphocytes	0.045	− 0.069	− 0.046	0.159

^*^p < 0.05, **p < 0.01

## Discussion

Our purpose in this work was to further the understanding of the nature of the relationship between cognitive status and age-related olfactory identification ability, as well as the predictive value of olfactory ability in the identification of subjects at risk of cognitive decline.

The results of this study indicate an age-related decline in olfactory identification ability and subjective intensity of odor perception. Olfactory identification declines with cognitive function, and the predictive power of olfactory identification scores for the risk of mild and severe cognitive impairment is approximately 80%. In addition, performance in odor identification is associated with impairment in episodic memory and executive functions.

In this study, the results indicate that age has a significant effect on general cognitive status. This is an undisputed fact in the scientific literature. Age is an indicator of risk for cognitive impairment. These results are in agreement with abundant studies indicating that aging is often accompanied by a decline in cognition, characterized by cognitive difficulties in memory, executive functions, learning ability, motor performance, and a generalized slowing of information processing [54–57].

In our study, we have three groups differentiated by their cognitive status measured from the MoCA: healthy controls, mild cognitive impairment (MCI), and severe cognitive impairment; the results indicate a clear association between age and general cognitive status, so that the mean age of the severe cognitive impairment group is higher than that of the MCI group and higher than that of the healthy control group, and the age of the MCI group is higher than that of the healthy control group.

We found no relationship between general cognitive status and other variables studied, such as sex, toxic habits, or suffering or having suffered from allergies or COVID-19. In this sense, there are conflicting positions when considering whether these variables are associated with olfactory performance. Although there are evidences which points that women perform better in olfactory tests due to hormonal factors [10, 12, 58], there is open discussions about sex differences, as other studies refuse those results [17, 49, 59, 60]. Therefore, in the present study, we do not differentiate participants by sex. Likewise, no differences were found between smokers and non-smokers, similarly to other studies [2, 61], neither among frequent alcohol consumers [62, 63].

In addition, we found a relationship between general cognitive status and olfactory identification (recognition and subjective intensity procedure by Delgado-Losada [17]. Olfactory identification decreases with cognitive function. Our results are consistent with other studies showing the association between cognitive and olfactory functions [64]. There is no evidence to establish an interaction effect between age and olfactory performance, so the effect of olfactory identification on cognitive function is maintained across age groups.

Our results provide further evidence for the effect of age on olfactory ability, since, as can be seen in Table 1B, we found an age-associated decline in identification ability. It has been argued that the effects of age on olfaction can be explained by the effects of cognitive decline, and not by age or age-related hazards affecting the age [38]. Although other authors point out that age per se may not explain presbyosmia (age-associated olfactory dysfunction) since the decline in olfactory function with healthy aging appears to be much less than what has been observed so far [65, 66].

Studies generally describe the onset of the general decline in identification ability around the sixth decade of life, and are more pronounced from the seventh decade onward. In older adults without cognitive impairment, age correlates inversely with odor identification test scores [8, 12]. In older adults without cognitive impairment, age correlates inversely with odor identification test scores [32, 67]. Practically, this means that absolute scores on olfaction tests cannot be used to define abnormality, and age adjustment needs to be used [49].

Furthermore, in our results, age was inversely correlated with scores on the identification test and on the intensity at which odors are perceived. It is well established that aging is often accompanied by a decline in olfactory functioning, and while odor thresholds are less affected by age [12, 68, 69] while identification decreases significantly [70].

Olfactory identification is closely related to higher cognitive functions [71, 72]. Our results support those of other investigators who have reported that impairment of olfactory identification is strongly related to cognitive impairment [73–75]. Thus, in the studies of Wilson et al. reported that impairment of olfactory identification was significantly associated with the incidence of mild cognitive impairment [32].

The second objective of this study was to understand the predictive value of olfactory ability in the identification of subjects at risk of cognitive impairment, to distinguish between healthy participants (MoCA ≥ 26), at risk of mild impairment (MoCA 25–18), and at risk of severe impairment (MoCA ≤ 17). Two logistic regression models were estimated: the first aimed to classify healthy participants and those at risk of mild impairment, while the second aimed to classify between participants at risk of mild impairment and those at risk of severe impairment. The results of the first model indicate that age and olfactory identification (both recognition and subjective intensity scores) correctly classify 82.1% of individuals (AUC = 0.821) into controls/at risk of mild impairment. On the other hand, in the second model, it was found that age and olfactory identification (only recognition score in this case) correctly classify 72% of participants at risk of severe impairment/risk of mild impairment (AUC = 72%). In both models, the sensitivity is close to 0.8 (0.79 and 0.83, respectively), while in the second one, the specificity is 0.87. This indicates that the predictive ability of olfactory identification scores for the risk of mild (model 1) and severe (model 2) impairment is around 80%.

Our results are in line with the large number of studies published in recent years where lower scores or impairment on odor identification tests predict cognitive impairment years later [26, 27, 76–80]. A meta-analysis by Roalf et al. [35] concluded that olfactory impairment is present and a predictor in patients with MCI and a vast number of studies point to impairment in olfactory identification as a common factor in neurodegenerative diseases such as Alzheimer’s disease (AD) and Parkinson’s disease (PD) [77, 81, 82].

In our study, in addition to analyzing the relationship between the MoCA total score as a test to assess general cognitive status, we wanted to analyze in more detail the different cognitive areas it assesses and their relationship with olfactory identification. We found a statistically significant positive relationship between olfactory identification (recognition score) and MoCA episodic and conceptual thinking memory scores. Our results are in line with studies that have been suggesting for decades that odor identification requires episodic memory and executive functions, and its dysfunction may represent a generalized cognitive impairment [83, 84]. Thus, although decidedly noteworthy, our finding that odor identification performance predicts impairment in episodic memory and executive functions is not entirely novel [78, 85, 86] is further evidence in favor of the relationship between olfaction and cognition.

The mechanisms underlying the association between olfaction and cognition have been extensively examined by both psychophysical and neuroanatomical studies [87–89]. For example, psychophysical studies have revealed that olfactory identification was significantly associated with memory, implying that the two may share some cognitive domains [38, 59, 68]. The change in olfactory identification has been strongly associated with pathological changes in medial temporal lobe structures [90, 91]. These studies strongly implicate a primary role of olfactory identification dysfunction as an indicator of cognitive impairment.

Finally, as discussed in the introduction, an impact in olfactory function may alter be observed on ADLs, motor capabilities, nutrition, or personal hygiene [5, 6]. Functional capacity involves physical and cognitive functions related to the ability to perform activities of daily living without assistance, and is the main factor determining independence and quality of life in older adults [92]. Therefore, we applied a correlation analysis between olfactory identification scores, cognitive performance and functional capacity indexes (Barthel and FAC indexes), and health status (lymphocytes, cholesterol, vitamin D, vitamin B12 and albumin). The results in this analysis show statistically significant positive relationships between olfactory identification (recognition score) and the Barthel Index of ADLs, as well as cognitive performance (MoCA) with this index. Therefore, this result evidences how olfactory ability may affect the performance of functional activities in the elderly. Non-significant relationships (*r* = 0.195) were found between olfactory identification (recognition score) and FAC Index, which measures motor capabilities. Although not statistically significant, this correlation might be an interesting future research question, as some studies have shown that olfaction is related to motor functions and gait in age-related cognitive decline [93]. No significant relationships were found with nutritional variables, so further studies are required regarding this topic.

The present study has some limitations that should be taken into account when interpreting the results obtained. Although the sample size may not be considered large, it is in line with other similar studies [79, 94]. Participants were divided into groups based on the results obtained from the MoCA which is a valid and reliable measure of cognitive performance, and although it cannot be considered a substitute for formal clinical assessment of participants, it has demonstrated a high sensitivity to cognitive impairment [42].

Furthermore, although this study observed a significant association between olfactory identification and cognitive impairment, it did not examine the underlying mechanisms involved in such a distinctive effect for odor identification compared to other olfactory functions. The MoCA subscores cannot substitute for an adequate assessment of cognitive function, but they provide descriptive and approximate measures of the cognitive subdomains analyzed. With this in mind, the results of this study with MoCA subscores support further studies with a full cognitive assessment that delve deeper into the mechanisms underlying these results. Even more, it would have been of interest to have information on polymerase chain reaction (PCR) to detect the ε4 allele of apolipoprotein E (ApoE ε4) and to be able to investigate olfactory identification and ApoE ε4 in the three groups of participants. The ɛ4 allele of the apolipoprotein gene is a genetic risk factor for late onset dementia of Alzheimer’s type, which is characterized by loss of both memory and olfactory functions. It would also be of interest in the future to be able to follow them over time and to be able to establish conclusions on the usefulness of olfactory identification as a predictor of conversion from healthy participants to MCI, and from MCI to severe cognitive impairment.

In conclusion, the association between olfactory function and cognitive impairment established in this study provides further evidence in support of including an olfactory assessment along with other neuropsychological measures in standard health examinations in clinical practice for older adults. We want to highlight our contribution for our results are especially important as they reveal that odor identification is associated with measures of memory and abstraction.

This is of clinical importance as, until now, memory performance is the best-known measure of cognitive impairment and incipient neurodegenerative disease. The results underscore the need to further study changes in olfactory identification as a useful measure for selecting/stratifying patients in treatment trials of cognitively impaired patients or prevention trials in cognitively intact individuals, because olfactory deficits may predict cognitive impairment. In addition, studies should include brain imaging data to investigate possible underlying structural and functional factors related to the olfactory changes observed in the present study.

## Supplementary Information

Below is the link to the electronic supplementary material.

Supplementary figure 1. (PNG 184 kb)

High resolution image (TIF 87.4 kb)

Supplementary file2 (XLSX 10.0 kb)

## Data Availability

Data at individual level is available upon request to corresponding author.
