# Supplementary figures and images for: Could olfactory identification be a prognostic factor in detecting cognitive impairment risk in the elderly?
Source: GeroScience. 2023 Apr 21;45(3):2011–25. doi: 10.1007/s11357-023-00779-5 (PMC10119830; doi:10.1007/s11357-023-00779-5)

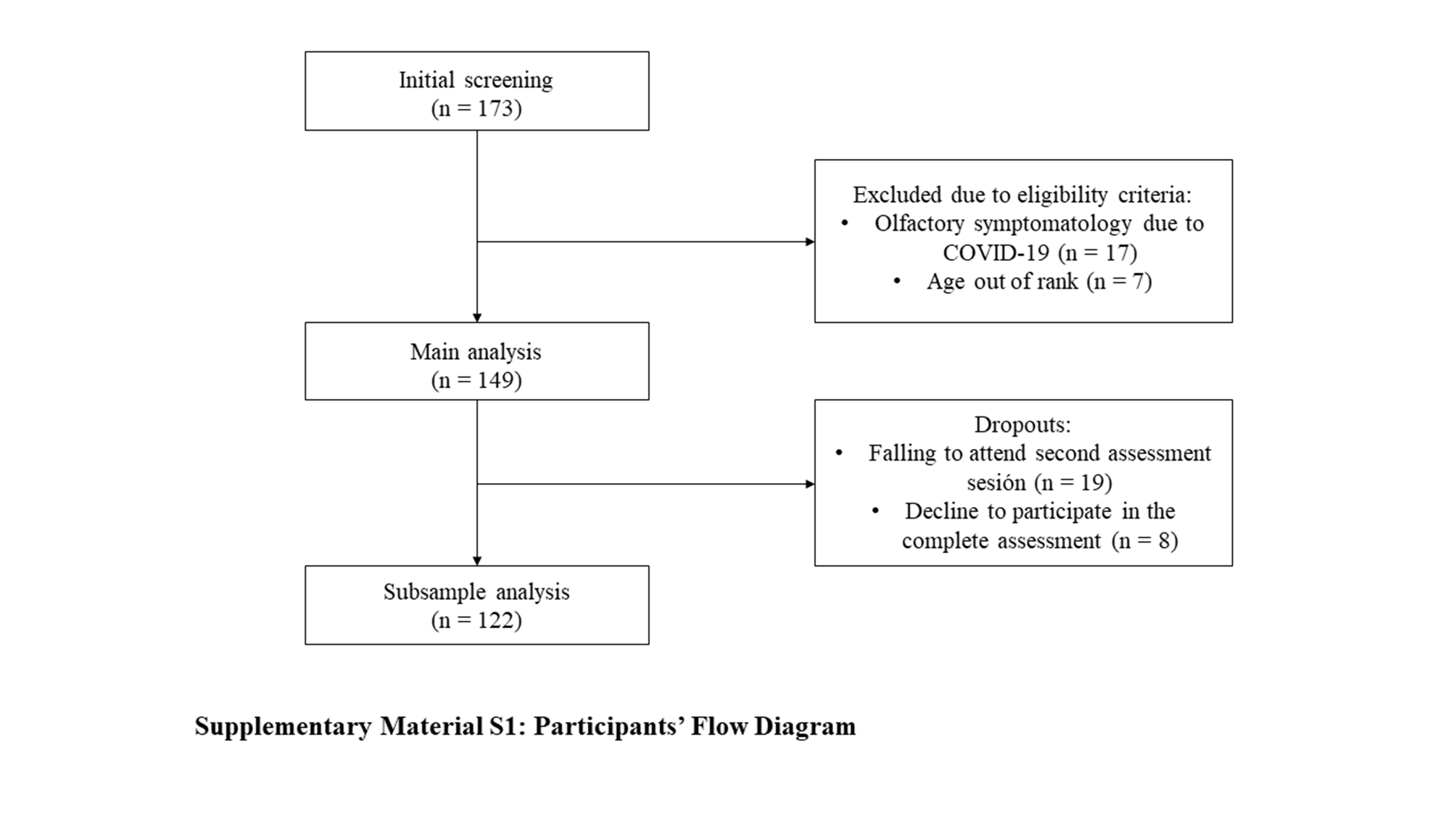

Supplement: Supplementary file 1 — (PNG 184 kb) [file 11357_2023_779_Fig3_ESM.png]

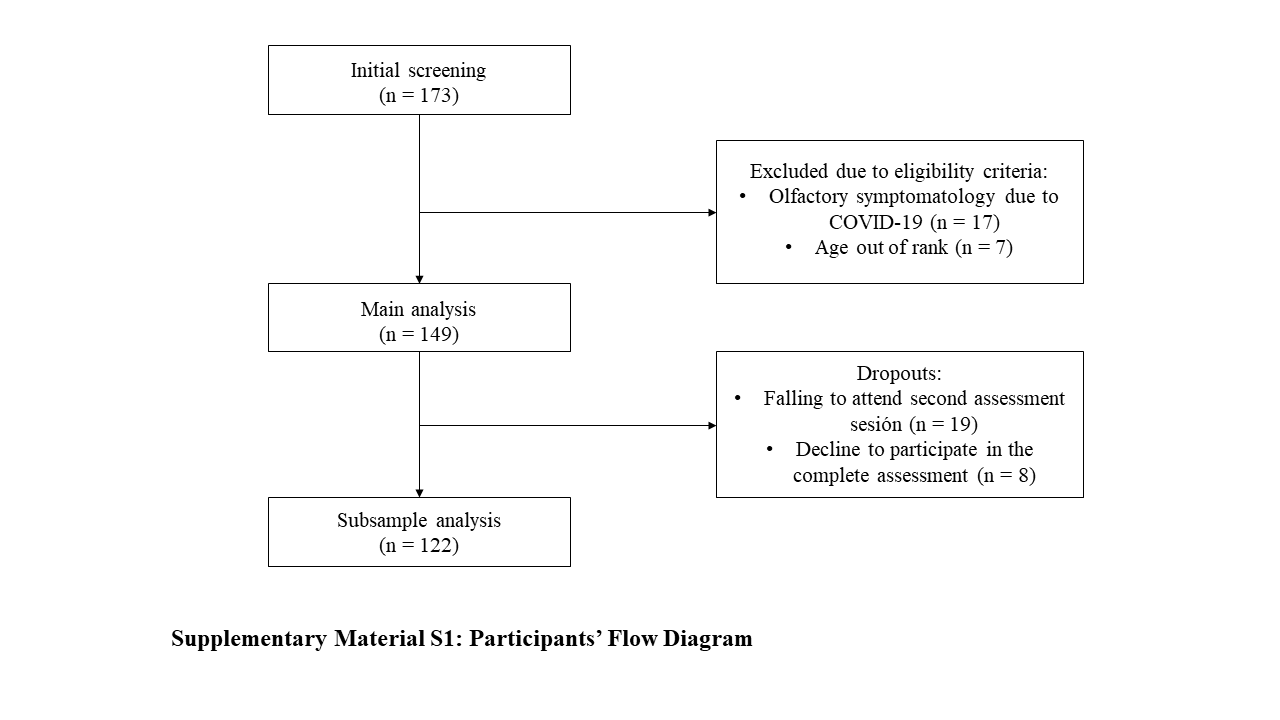

Supplement: Supplementary file 2 — High resolution image (TIF 87.4 kb) [file 11357_2023_779_MOESM1_ESM.tif]
